# Sociocultural Differences in Accepting Technology for Older Adults Between South Korea and the United States

**DOI:** 10.1093/geroni/igab046.2108

**Published:** 2021-12-17

**Authors:** Jeungkun Kim, Suk-Young Kang

**Affiliations:** 1 Kangnam University, Yongin-si, Kyonggi-do, Republic of Korea; 2 Binghamton University, State University of New York, Binghamton, New York, United States

## Abstract

In recent years, Western-originated technology products for older adults are rapidly spreading in Korea, but discussions on technology acceptance taking into account the socio-cultural characteristics of older adults in Korea are relatively insufficient. The purpose of this study is to analyze the influence of the socio-cultural characteristics of Korean older adults on their intention to use technology compared to the United States. Due to Covid-19, a telephone and non-face-to-face survey was conducted for older adults aged 65-95 residing in New York State in the U.S. and the metropolitan area of South ​​Korea from September 2020 to January 2021(N=155 in South Korea, N=180 in the U.S.).In this study, the expanded technology acceptance model for older adults was conceptualized, and socio-cultural factors were used as mediators or modulators. Results show that Korean older adults had higher expectations that technology use would have a positive impact on their lives, and their product purchase intentions were higher than those in the United States(p<0.001). The main reason was that Korean older adults were less anxious about the leakage of personal information, had higher national trust and were relatively less resistant to robots than American older adults. In addition, Korean older adults were more confident that they could receive help in case of problems with technology products than their counterparts in the United States. This study suggests practical and policy alternatives for securing technology acceptance of older adults, taking into account the social and cultural factors of Korea and the United States.

